# Effect of Filler Synergy and Cast Film Extrusion Parameters on Extrudability and Direction-Dependent Conductivity of PVDF/Carbon Nanotube/Carbon Black Composites

**DOI:** 10.3390/polym12122992

**Published:** 2020-12-15

**Authors:** Beate Krause, Karina Kunz, Bernd Kretzschmar, Ines Kühnert, Petra Pötschke

**Affiliations:** Leibniz-Institut für Polymerforschung Dresden e.V. (IPF), Hohe Str. 6, 01069 Dresden, Germany; krause-beate@ipfdd.de (B.K.); karinakunz2015@gmail.com (K.K.); bkretzsch@online.de (B.K.); kuehnert@ipfdd.de (I.K.)

**Keywords:** polymer composites, hybrid filler systems, electrical conductivity, film extrusion

## Abstract

In the present study, melt-mixed composites based of poly (vinylidene fluoride) (PVDF) and fillers with different aspect ratios (carbon nanotubes (CNTs), carbon black (CB)) and their mixtures in composites were investigated whereby compression-molded plates were compared with melt-extruded films. The processing-related orientation of CNTs with a high aspect ratio leads to direction-dependent electrical and mechanical properties, which can be reduced by using mixed filler systems with the low aspect ratio CB. An upscaling of melt mixing from small scale to laboratory scale was carried out. From extruded materials, films were prepared down to a thickness of 50 µm by cast film extrusion under variation of the processing parameters. By combining CB and CNTs in PVDF, especially the electrical conductivity through the film could be increased compared to PVDF/CNT composites due to additional contact points in the sample thickness. The alignment of the fillers in the two directions within the films was deduced from the differences in electrical and mechanical film properties, which showed higher values in the extrusion direction than perpendicular to it.

## 1. Introduction

In recent years, electrically conductive polymer composite (CPC) materials have aroused increasing interest worldwide due to their unique property combination, such as enhanced mechanical properties and the adjustability of electrical conductivity [1,2]. In particular, carbon nanotube (CNT) modified polymer composites are very attractive, as they can combine the extraordinary properties of the CNTs with the advantageous properties of polymers such as low weight, corrosion resistance, easy capability, and low costs. Thus, composites with high stiffness, high strength, and high electrical conductivity can be achieved at low filler concentrations. On a large scale, the most common production method is melt processing. A good dispersion of fillers, especially of CNTs, in the thermoplastic matrix during the melt mixing step is a challenge and has been described in many publications including studies on small-scale and large-scale compounding [3,4,5,6,7,8,9,10]. Next to structural parameters of the CNT material, processing parameters such as extrusion temperature, screw speed, throughput, and the way of filler feeding play important roles on the achievable dispersion [6,11,12]. The shaping of the materials can take place via various processes, such as compression molding, injection molding, shape extrusion (such as tubes), film extrusion (cast or blown), 3D printing, and various coating processes. Cast film extrusion is an industrial method offering the possibility of continuous production of self-standing films [13,14,15]. During the cast film extrusion, the polymer melt exiting a single-screw extruder through a slit die is stretched in air and cooled on a smooth metallic surface of a thermoregulated chill-roll. After that, the film is wound up. The stretching stress is dependent on the take-off velocity. As the main processing parameters, the roll temperature, the take-off velocity, and the cooling during taking-off influence the final microstructure already of unfilled polymers. There are only a few scientific publications [13,14,15] that deal with influencing factors during the melt-shaping of polymer films, e.g., for polycarbonate [13] and polypropylene [14]. For composites containing CNTs, the phenomenon of a strong alignment of CNTs in the extrusion direction was discussed on the example of blow-molded high-density polyethylene/multiwalled carbon nanotube films, which resulted in enhanced mechanical properties in this direction [16]. No publications were found concerning the direction-dependent electrical properties of thin cast-film extruded CNT containing composites.

One of the most important parameters both in the compounding step as in the film extrusion is the melt viscosity of the composite, which is mainly influenced by the molecular weight of the polymer matrix, the processing temperature, and the filler concentration. The melt viscosity of the polymer matrix influences the shear stresses acting on the CNT agglomerates during the extrusion-mixing step [16]. The shear stress is responsible for both the filler dispersion and shortening of CNTs [17,18,19]. With increasing shear stresses, a lower number of remaining CNT agglomerates can be expected, but at the same time, the more pronounced CNT shortening can lead to increased electrical percolation concentration [19,20]. A balance is aimed for between these different trends.

The shear stresses during melt extrusion lead also to filler orientation, while the melt is being let out of the die, which is reflected in the electrical and mechanical composite properties [21,22,23,24]. During extrusion, fillers with a high aspect ratio, such as CNTs, are aligned along the extrusion direction [25]. In film extrusion, these fillers are oriented in the film plane. This results in the effect that at low filler concentrations, a film can be conductive in plane but not through the film thickness [26]. This can only be counteracted by using a higher filler content. In contrast, spherical or low aspect ratio fillers, such as carbon black, can hardly be oriented. Therefore, the idea of combining high-aspect fillers such as CNTs with spherical fillers such as carbon black (CB) seems to be an effective strategy to minimize the filler orientation by maintaining high electrical conductivity.

The effects of combination of high (CNT) and low aspect ratio fillers (CB) in composites on electrical and mechanical properties of thermoplastic composites has already been described by many authors [27,28,29,30,31,32,33,34,35,36]. Mixed filler systems can lead to lower percolation thresholds compared to the single systems [28,30] or to higher electrical conductivity at the same CNT content [33] or the same overall filler content [34]. The formation of a combined electrical network of both fillers was reported [27,29,31,35,36,37]. With a mixed filler system of CNT and CB, it should be possible to increase especially the conductivity through film compared to polymer/CNT composites.

In the present study, melt mixed poly (vinylidene fluoride)-based composites filled with hybrid filler systems of carbon nanotubes and carbon black were developed using a small-scale microcompounder. The composite preparation was upscaled to laboratory extrusion, whereas direct compounding and masterbatch dilution were compared. The aim was to obtain composites free of agglomerates in the microscale, which represents a precondition for the subsequent film extrusion step, in which larger agglomerates would result in holes in the desired thin films with target thicknesses in the range of 50–100 µm. The film extrusion was performed on these composites under variations of processing temperature and take-off velocity. Direction-dependent mechanical and electrical properties were characterized on compression-molded plates and films. For example, such films can be used as current collector films that replace aluminum foils in bipolar battery architectures in lithium-ion batteries, as shown in [24].

## 2. Materials and Methods

### 2.1. Materials

**Figure 1:**
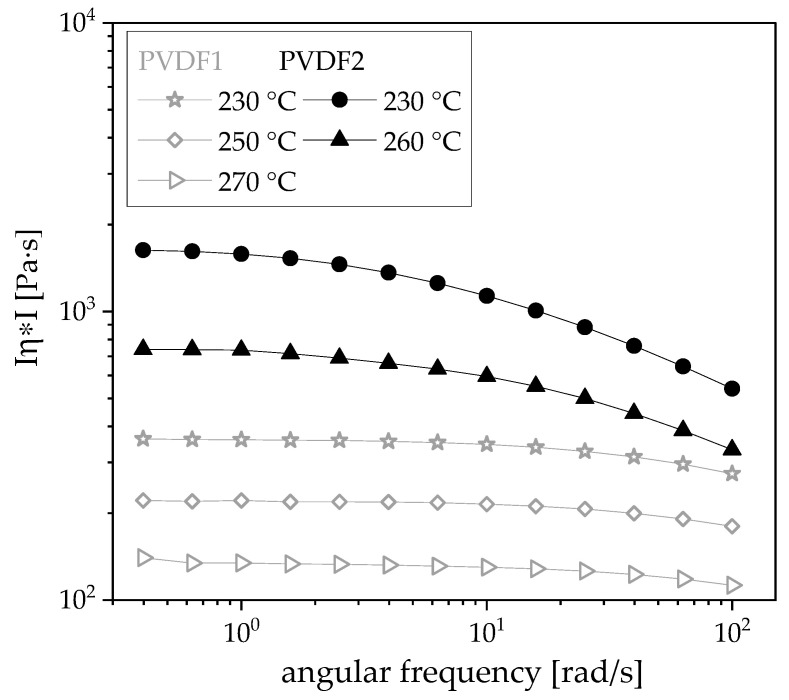
Complex melt viscosity Iη*I in dependence on the angular frequency of pure poly (vinylidene fluoride) (PVDF), PVDF1 and PVDF2 measured at different temperatures.

Two commercially available poly (vinylidene fluoride) (PVDF) grades were applied, namely Solef1006 (Solvay, Lyon, France, PVDF1) with a melt flow index of 40 g/10 min (230 °C, 2.16 kg) and Kynar720 (Arkema, Colombes Cedex, France, PVDF2) with a melt flow index of 5–29 g/10 min (230 °C, 5.0 kg). Rheological measurements were performed on pure PVDF materials whereby higher levels of viscosity (see Figure 1) and elasticity (see Figures S1 and S3) were found for PVDF2 compared to PVDF1.

As electrically conductive fillers, mixtures of branched multi-walled carbon nanotubes (b-MWCNTs) and carbon black (CB) were chosen. The b-MWCNT “CNS flakes” (Applied NanoStructured Solutions LLC, Baltimore, MD, USA) have a diameter of 14 ± 4 nm and length of ≈70 µm (aspect ratio ≈5000) [38] and are coated with 3 wt % poly (ethylene) glycol. The CB is a highly structured type of the grade Ketjenblack^®^ EC600JD (Akzonobel, Cologne, Germany) with a surface area of 1200 m^2^/g and a primary particle size d_50_ of 34 nm (according to the supplier). Scanning electron microscopy images of both fillers are shown in Kunz et al. [26].

### 2.2. Composite Preparation

Melt mixing experiments in small scale were carried out using a conical twin-screw microcompounder DSM15 (Xplore, Sittard, The Netherlands) having a volume of 15 cm^3^. The fillers and polymer granules were dried for 1 h at 80 °C in a vacuum oven and then mixed for 10 min at 210 °C and a rotation speed of 200 rpm. The resulting strands were cut in small pieces and after drying again compression molded to circular plates with a thickness of ≈300 µm and a diameter of 60 mm, using the hot press PW40EH (Otto Paul Weber GmbH, Remshalden, Germany) at 200 °C, pressure of 50 kN, and compression time of 2.5 min.

Melt compounding at a larger scale was performed using a laboratory twin-screw extruder ZE 25 (KraussMaffei Berstorff GmbH, Hannover, Germany) with a screw with an length/diameter ratio of 48 [39]. The pre-mixed PVDF powder with carbon fillers was compounded at a temperature of 210–230 °C, a rotation speed of 200 rpm, and a throughput of 5 or 10 kg/h. Composites were prepared by direct compounding (DC), masterbatch dilution (MBD) or for homogenization, the composite was extruded again under the same conditions (double extrusion, DE). The extruded strands were granulated into approximately 2 mm diameter pellets.

Cast film extrusion was performed with these composite granules using a 30 mm single-screw extruder (DAVO GmbH, today Polyrema KG, Troisdorf, Germany) in combination with a cast film line (Dr. Collin GmbH, Maitenbeth, Germany). The width of the flat die was 30 cm, the gap width was set to 100 µm, and the mass temperature was varied between 225 and 290 °C. The take-off velocity was varied between 2.5 and 7.5 m/min. About 50 m of films were produced under stabilized extrusion conditions.

### 2.3. Material Characterization

The complex melt viscosity Iη*I of PVDF and the composite materials was measured with an ARES oscillation rheometer (TA Instruments, New Castle, DE, USA). The oscillation measurements were carried out under nitrogen atmosphere in the temperature range 230–290 °C, with a parallel plate geometry (diameter 25 mm, gap approximately 1 mm). Dynamic frequency sweeps (strain 5%) with increasing and decreasing frequency (between 0.4 and 100 rad/s) were used, whereby the second sweep was used for interpretation.

The morphological characterization of the fillers and composite materials was performed by scanning electron microscopy (SEM) on surfaces and cryofractured cross-sections of extruded films with a Carl Zeiss Ultra plus microscope (Carl Zeiss Microscopy, Jena, Germany) at 20 kV acceleration voltage and the Inlens detector using the charge contrast imaging mode (CCI).

The electrical volume resistivity of the composites was measured using two different configurations, depending on the resistivity of the samples. For samples with resistivities above 1E7 Ohm-cm, the compression-molded plates or extruded films were characterized using a Keithley 8009 Resistivity Test Fixture combined with a electrometer E6517A (Keithley Instruments, Cleveland, OH, USA). For samples with lower resistivity, the TWM1 device was used, which is able to measure the in-plane and through-plane directions separately [26]. The following terms are used for the measured directions in the extruded films: **x** indicates in plane and in the extrusion direction, **y** indicates in plane and perpendicular to the extrusion direction, **z** indicates through plane. For compression-molded plates, no alignment within the plane is expected, so that x and y are identical. Furthermore, different quotients of the electrical conductivities σ are calculated: σ_x/z_ gives the quotient of the value of the x-direction to that in the z-direction, σ_y/z_ gives the quotient of the value of the y-direction to that in the z-direction, σ_x=y/z_ gives for compression-molded plates the quotient of the value of the x=y direction to that in the z-direction, and σ_x/y_ gives the quotient of the value of the x-direction to that in the x-direction.

Tensile tests for the determination of mechanical properties were performed with a tensile universal testing machine Z010 (ZwickRoell, Ulm, Germany) based on strips of films (length 115 mm, width 10 mm) with their length axis in the film extrusion direction (x) or perpendicular to it (y) and a displacement rate of 5 mm/min (according to DIN 53504/1A/5). The values given are mean values and standard deviations of at least five measurements.

## 3. Results and Discussion

### 3.1. Comparison of Single and Hybrid Filler Systems in PVDF Composites

**Figure 2:**
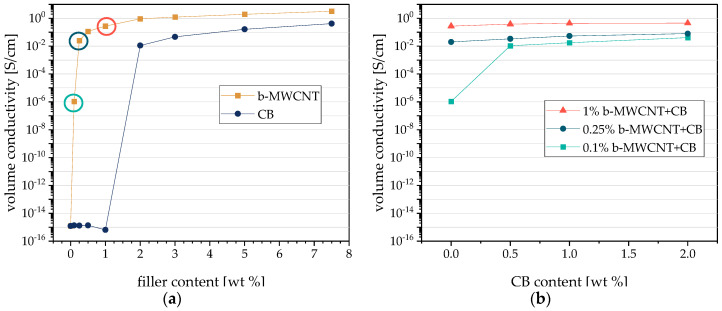
Volume in-plane conductivity of poly (vinylidene fluoride) (PVDF) composites with different fillers (**a**) branched multi-walled carbon nanotubes (b-MWCNT), carbon black (CB), (**b**) mixed filler systems of b-MWCNT and CB in PVDF1. The circles in (**a**) mark the concentrations of b-MWCNTs used in (**b**).

The percolation curves of PVDF1 composites filled with b-MWCNT or CB as measured in plane on compression-molded plates prepared from composites mixed in small-scale are shown in Figure 2a. As expected, the percolation threshold for PVDF1/b-MWCNT composites at 0.1 wt % b-MWCNT is much lower compared to that of PVDF1/CB composites at 1–2 wt % CB. This significant difference is due to the different shapes of b-MWCNT with a high aspect ratio compared to the more spherical CB aggregates. The high aspect ratio CNTs enable the formation of an electrically conductive network at lower contents.

The study of hybrid filler systems in PVDF1 composites was performed at constant b-MWCNT contents under addition of 0.5–2.0 wt % CB (Figure 2b). At the b-MWCNT level of 0.1 wt % b-MWCNT, which is the percolation threshold for this system, the incorporation of 0.5 wt % CB leads to an increase in conductivity by 4 decades up to 1.06 × 10^−2^ S/cm, which is the same range as that for PVDF1/2 wt % CB and PVDF1/0.5 wt % b-MWCNT composites. It can be concluded that a synergistic network of both fillers occurred in the composite with 0.1 wt % b-MWCNT and 0.5 wt % CB, as 0.5 wt % CB alone is not enough to form a conductive network. If the composites contain 0.25 or 1.0 wt % b-MWCNT, the CB addition leads only to a marginal increase of conductivity; however, the values are up to one decade higher than with 0.1 wt % b-MWCNT.

### 3.2. Influence of the Preparation Procedure on the Electrical Conductivity: Direct Compounding (DC) vs. Masterbatch Dilution (MBD) and Microcompounder vs. Laboratory Extruder

**Figure 3:**
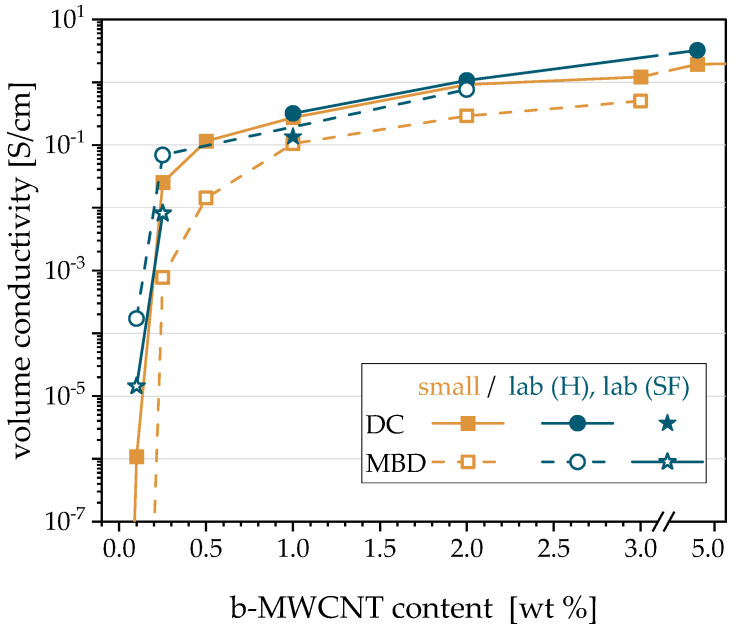
Electrical volume in-plane conductivity of PVDF1/b-MWCNT composites (compression-molded plates) prepared at small scale or laboratory scale (10 kg/h) and direct compounding (DC) or masterbatch dilution (MBD; small scale from 7.5 wt % to lower contents, lab scale from 5 wt % to 2 wt % and from 2 wt % to 0.1 and 0.25 wt %).

For the composite preparation using the microcompounder, a comparison of the in-plane electrical properties of compression-molded plates of PVDF1/b-MWCNT composites was carried out between the direct incorporation (DC) of b-MWCNTs and the masterbatch dilution (MBD) of a PVDF1/7.5 wt % b-MWCNT masterbatch. Former studies reported a lower electrical percolation threshold for composites prepared using direct compounding than for masterbatch dilution [40,41]. In addition, for the same CNT contents, the electrical conductivity values were found to be higher for direct incorporation than for masterbatch dilution. Both findings from the literature could be observed also in the present study (Figure 3). The percolation threshold of 0.1 wt % b-MWCNT found for composites prepared using direct compounding is slightly lower than that of 0.2 wt % for MBD. Using the MBD, the composite with 0.1 wt % CNT is not electrically conductive. The electrical conductivity values of composites prepared using DC were always higher than those of the composites prepared using MBD. The differences are particularly large, as the low CNT contents and can be mainly explained by a more severe nanotube shortening when using the twofold compounding in the MBD approach.

In a further step, composites were produced in larger scale using the laboratory extruder. Composites with 1, 2 and 5 wt % b-MWCNTs were prepared by DC. The electrical conductivities of their compression-molded plates were in the same range as those of the composites prepared using DC in the microcompounder (blue circles in Figure 3). The composite filled with 5 wt % was diluted to 2 wt % CNT content, and its electrical conductivity is comparable to that of the composite with 2 wt % produced by DC. Furthermore, the PVDF1/2 wt % b-MWCNT composite was further diluted to composites that contain 0.1 or 0.25 wt % b-MWCNTs. Their electrical conductivities (blue rings in Figure 3) are higher compared to the composites prepared using the microcompounder (DC and MBD), which is an indication that the laboratory extruder is slightly better suited to produce conductive composites. It also shows quite nicely that upscaling from the gram quantity of the material in microcompounding to the kilogram quantity in extrusion on a laboratory scale was successful and thus promising for further development into material production. In addition to the two different preparation methods (DC, MBD), the influence of the CNT dosage position (hopper (H) or side feeder (SF)) on the electrical properties of the composites was also investigated. In the experiments described so far, the CNTs or masterbatch were always dosed via the hopper. It was found that for both DC and MBD, the dosing of the fillers via the side feeder leads to significantly lower conductivities (filled and unfilled stars in Figure 3) compared to feeding in the hopper. Based on these results, it was decided that in all further experiments using the laboratory extruder, the dosing occurs via the hopper. In addition, further investigations were carried out using DC, as the higher efforts and energy connected with the MBD do not result in a noticeable advantage concerning the electrical conductivity.

Concerning the state of dispersion of b-MWCNT into PVDF, it was shown before by Krause et al. [38] using light microscopy on thin sections that the direct incorporation strategy leads to agglomerate-free composites. The process route via the masterbatch dilution was found in former studies to improve dispersion and resulted in a reduction of residual CNT agglomerates compared to direct incorporation [41]. Since agglomerate-free composites are already achieved using direct b-MWCNT incorporation, light microscopic examinations were dispensed within the present study, because a difference in the CNT dispersion was not expected.

### 3.3. Direction-Dependent Electrical Conductivity in Compression-Molded Plates and Extruded Films

So far, in-plane measurements on compression-molded plates were presented. This measurement direction follows the typical standards for electrical conductivity on moderately conductive samples. However, for many applications, the conductivity through the plane is also of interest and, in the case of extruded films, so is the orientation in and perpendicular to the extrusion direction.

Therefore, to gain more insight into the structure of the conductive filler network, direction-dependent measurements of electrical properties were performed on compression-molded plates as well as on extruded films. In order to investigate only the influence of the shaping process, compression molding and film extrusion were carried out with materials produced in the laboratory scale. As shown by Kunz et al. [26], quotients of the respective conductivity values in different measuring directions can be used to obtain information about the degree of filler orientation. It was reported that in compression-molded plates, CNTs were strongly orientated in plane having quotients of in-/through-plane conductivity σ_x=y/z_ between 1071 and 14, depending on the filler content. These values varied with CNT type and decreased with the higher distance of the actual filler content to the respective percolation threshold concentration.

Direction-dependent conductivity values as well as conductivity ratios between the different directions of PVDF1/b-MWCNT composites (based on [26]) and of hybrid filler systems are shown in Table 1 and Table 2. For extruded films containing CNTs only, the CNTs are strongly oriented in plane (x,y), resulting in lower conductivities in the z-direction (through-plane), as it was discussed in our earlier study. In the in-plane direction, the CNTs are oriented more along the extrusion direction (x-direction) than transversely (y-direction). In contrast, PVDF/CB composites show only conductivity ratio values (σ_x=y/z_, σ_x/z_, σ_y/z_) of 1-6, which indicates that no significant orientation can be measured for both compression-molded plates and extruded films (Table 1 and Table 2).

In the hybrid filler systems, the addition of increasing CB contents to PVDF with 1 wt % b-MWCNT leads to increasing the electrical conductivity in all measurement directions for both compression-molded plates and extruded films (Table 1). Only the sample with 1 wt % CB addition shows a very slight decrease in all conductivity values, whereas through the plane, this film shows a higher value than the film with 1 wt % b-MWCNT. The comparison of the different σ quotients (σ_x/z_, σ_y/z_, σ_x/y_) shows a decrease of these values with increasing CB content, which indicates a less pronounced overall filler orientation in the combined network of CNTs and CB particles (Table 2). It is assumed that the orientation of CNTs is only slightly influenced by the CB addition, as the CNT orientation within the shear stress direction is induced by their high aspect ratio. It can be concluded that the CB particles are arranged between the CNTs and thus compensate for the strong orientation of the CNT conductive network. The comparison of electrical conductivity values of extruded films of PVDF1/1 wt % b-MWCNT and PVDF1/1 wt % b-MWCNT + 4 wt% CB shows the discussed effect impressively. The electrical conductivity in the x- or y-direction increases to the 2-fold or 4-fold value. However, in the through-plane direction (z), the σ value increase to the 22-fold value of the composite with CNTs only. Furthermore, the z-conductivities of PVDF1/1 wt % b-MWCNT + 4 wt % CB and PVDF1/4 wt % CB are almost the same with 6.9 × 10^−2^ S/cm and 6.1 × 10^−2^ S/cm, respectively, while the σz of PVDF/1 wt % b-MWCNT is significantly lower at 3.2 × 10^−3^ S/cm. From this, it can be concluded that the arrangement of the CB particles is the main reason for the higher σ_z_ value.

The influence of the shaping process on the electrical conductivity can be discussed using the quotients of the conductivities when considering the same direction (σ_z plate/film_, σ_x = y plate/x film_, σ_x = y plate/y film_), as shown in Table 1. For the PVDF/CB composites, the quotients in the x- and y-directions are 1, which indicates no influence of the shaping process on the electrical properties in these directions. Interestingly, the quotient σ_z plate/film_ is 0.5, meaning that the plate has a lower through-plane conductivity than the film. It can be concluded that the higher and shear and elongational stresses during film extrusion lead to a more developed network through the film than in the compression-molded plate. For PVDF/1 wt % b-MWCNT + CB composites, in the x-direction, the quotient σ_x = y plate/x film_ has, independent of the CB content, the value of 1, which indicates that the shaping process does again not play a role for electrical conductivity in the plane. The lower conductivity of extruded films in the y-direction than in the x-direction is reflected in the quotient σ_x = y plate/y film_, which varies between 3 and 7 and decreases with increasing CB content. This means that a higher CB content leads to more homogeneous electrical properties in the in-plane direction of the extruded films. The ratio of z-conductivity σ_z plate/film_ decreases with increasing CB content in PVDF/1 wt % b-MWCNT composites. This means that higher conductivities were achieved through the film during film extrusion than after compression molding. These findings correlate with results of σ_z plate/film_ for PVDF/CB composites described above. It can be summarized that film extrusion enables higher z-conductivities compared to the compression-molding process, while the in-plane conductivities (x, y) are higher in compression-molded specimens.

Regarding the electrical conductivity quotients of σ in plane (x, y) divided by σ_z_, clear tendencies are visible (Table 2). In compression-molded plates of PVDF1/1 wt % CNT and PVDF1/1 wt % CNT+CB composites, the σ_x = y/z_ is between 43 and 58, which indicates a filler orientation in the in-plane direction (x = y). The low quotient of 5-6 for the PVDF1/CB composite means that there is very little orientation in the plane of compression-molded plates. With higher CNT content, σ_x = y/z_ rises from 56 to 84, indicating an even stronger CNT orientation in the in-plane direction (x = y). When comparing the composites with CNT, CB, and hybrid filler systems, it becomes clear that if CNTs are present, the conductivities in plane are significantly higher than through the specimen. This leads to the conclusion that there is a strong preference for an in-plane direction orientation of the overall network.

In extruded films, considering the conductivity differences between the two in-plane directions and the z-direction, a higher filler orientation through the films can be concluded for CNTs compared to CB. This can be assigned from higher quotients σ_x/z_ and σ_y/z_ (≈150 and ≈41, respectively) for CNTs than for CB (3-5 and 3-4, respectively). The addition of CB to CNTs leads to a significant decrease of quotients σ_x/z_ and σ_y/z_, indicating that the CB particles form bridges between the CNTs and form new conductive pathways. With increasing CB content in the composite, the filler orientation decreases.

In the extruded films, the filler orientation in the plane, as deduced from the quotient of electrical conductivity σ_x/y_, can be seen clearly. The composites containing b-MWCNTs show a value of 4 for the quotient σ_x/y_, indicating that the b-MWCNTs are oriented in the extrusion direction (x). In comparison, PVDF1/CB composites show a value of 1, indicating no such orientation. For the hybrid filler systems, this quotient lies with 2 and 3 between the values for CNTs and CB, which indicates a slight reduction of orientation in the x-direction due to the CB addition.

**Figure 4:**
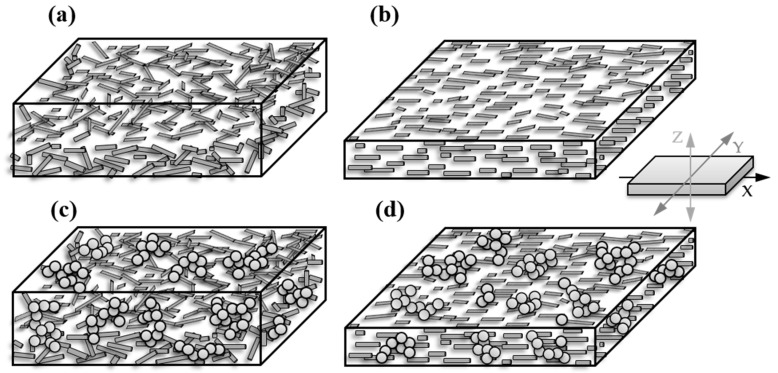
Schematic illustration of filler network in polymer matric: (**a**) compression-molded plate with carbon nanotubes (CNTs), (**b**) extruded film with CNTs, (**c**) compression-molded plate with CNTs and CB, (**d**) extruded film with CNTs and CB.

A schematic illustration of the assumed arrangement and orientation of the fillers CNTs and CBs is shown in Figure 4. Whereas in thicker compression-molded plates (Figure 4a) the CNTs are slightly aligned in plane parallel to the plate surfaces, no orientation within this plane is seen. In extruded films, a stronger alignment in plane and a strong orientation in the extrusion direction (x) is seen. When using hybrid filler systems of CNTs and CB, the CB aggregates are expected to bridge the nanotube connection points, especially in the z-direction.

**Figure 5:**
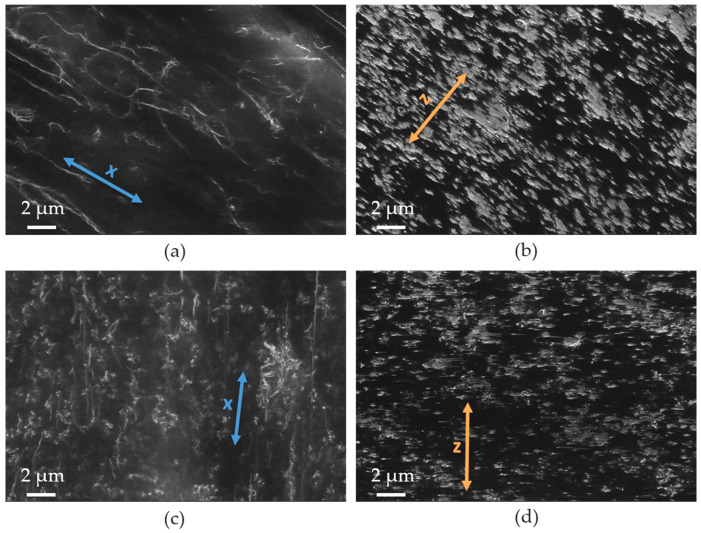
SEM-charge contrast imaging mode (CCI) images of extruded PVDF1 films: (**a**,**b**) PVDF1/2 wt % b-MWCNT, (**c**,**d**) PVDF1/1 wt % b-MWCNT + 1 wt % CB, (**a**,**c**) surface, arrows indicate the x-direction and alignment direction of CNTs, (**b**,**d**) cross-section, arrows indicate the z-direction of the film, the CNTs and CB aggregates are aligned perpendicular to the z-direction.

The SEM study using the charge contrast imaging mode (CCI) was applied to illustrate the electrical conductive network inside the extruded film. Due to the smooth surface of the films, effects caused by surface topography can be excluded. The CNTs can be seen as light gray lines and the CB particles can be seen as light gray spheres. Figure 5a,c shows the surface of PVDF1/2 wt % b-MWCNT and PVDF1/1 wt % b-MWCNT + 1 wt % CB composites. It can be clearly seen that the conductive network is much more oriented when only CNTs are contained compared to the mixture of CNTs and CBs. In the images taken in the cross-section, a clearer alignment can be seen for the PVDF/CNT composite than for the composite with the hybrid filler system (Figure 5b,d).

### 3.4. Mechanical Properties of Extruded Films

Some mechanical properties of the extruded films were characterized to determine their suitability for possible applications and their requirements (Table 3). For this purpose, the different composites were extruded to films with a thickness of 100 µm. This was achieved by adjusting the take-off velocity at 2.75 m/min. For PVDF1/1 wt % b-MWCNT + 3 wt % CB composite, the increase of the take-off velocity up to 3.25 m/min resulted in a thickness of 65 µm.

For all films, a strong direction dependency of tensile test results was found. Especially for the pure PVDF film, the stress–strain behavior was significantly different. Whereas the stress–strain curve in the extrusion direction shows a clear yield point at 5% strain and the strain at break is between 60 and more than 100%, the results in the y-direction show an increase in stress up to the maximum value of similar magnitude of ca. 48 MPa, at which the break occurs at much lower elongations (ca. 5%). The latter behavior with increase of stress and strain up to the breaking point was found for all PVDF1 composites. The addition of 2 wt % b-MWCNTs to PVDF results in an increase in modulus; however, it is only in the extrusion direction, whereas in the perpendicular direction, a decrease is found. This decrease is compensated by the addition of CB, except for the sample with only 60 μm thickness. For all composites, the tensile strength is higher in the extrusion direction as compared to perpendicular to it. In addition, the E-modulus has the tendency to be higher in the extrusion direction. The elongation at break is typically also slightly higher in that direction.

The comparison to the results of the thinner film shows lower values of all characteristic values of the stress–strain curve. This may be explained by the higher probability that small inhomogeneities result in an earlier break during the testing, which is also supported by the relatively high standard deviations.

The direction dependence of the mechanical properties is also expected to be related to the orientation of the filler networks. The orientations are higher in the extrusion direction, which is reflected in a tendency toward higher values in this direction. Crystallinity differences between the different films do not seem to have a main influence, as the melting enthalpies of the different composite films are almost the same (Table S1). The filler addition results in a slight decrease as compared to pure PVDF films, even if the crystallization temperatures are slightly enhanced by the filler addition.

### 3.5. Variation of Film Extrusion Process Conditions

As prerequisites for the extrudability of a thin film, a homogeneous filler dispersion and distribution as well as a suitable melt viscosity of the composite material at the given process conditions are required. If the filler dispersion is not good enough, small agglomerates may lead to defect spots that can result in cracks in the film during the extrusion process. If the melt viscosity is too high, the flowability of the melt into the slit die and a homogeneous feeding may not be guaranteed. In this context, also, melt elasticity may play a role. It is well-known that the addition of fillers reduces the pseudoplastic behavior and enhances both viscosity and elasticity. Therefore, the filler content must be optimized in the direction of using the smallest possible quantities of filler; however, these have to be sufficient for the required conductivity values.

In our study, in order to disperse the fillers in the composite without residual agglomerates, direct incorporation and masterbatch dilution were compared (see Section 3.2). Even if no visible agglomerates could be detected using light microscopy, the first films prepared using direct incorporation showed small holes, which were obviously due to filler agglomerates of smaller size. Therefore, the composites were homogenized under the same conditions in a second compounding step (double extrusion, DE). This enabled the production of much more uniform films. The optimization of melt viscosity during film extrusion was done by variation of the extrusion temperature. At the beginning, the film extrusion was performed at 225 °C, which is similar to the compounding temperature during composite preparation. However, only films down to 100 µm could be produced. At higher take-off velocities, holes became visible in the film, and the film tore. By increasing the temperature to 260–270 °C (PVDF1) or 280–290 °C (PVDF2), the take-off velocity could be increased so that films with only 50 µm could be produced.

**Figure 6:**
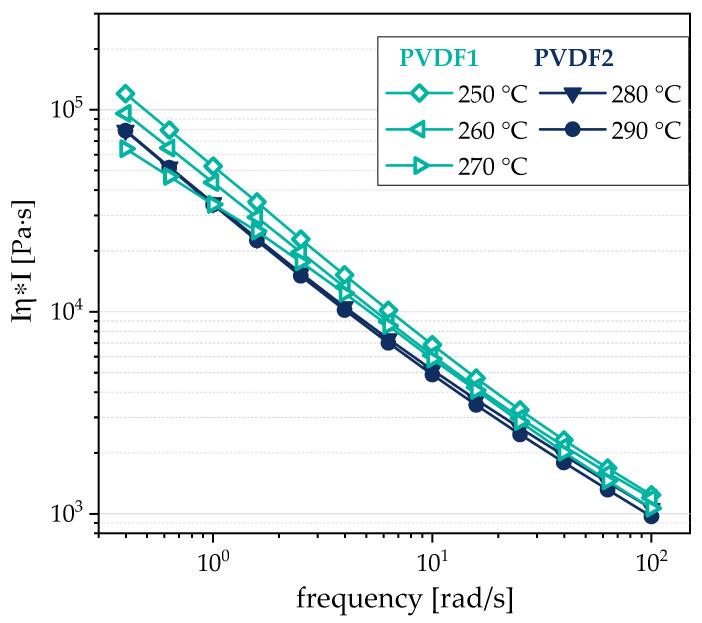
Complex melt viscosity Iη*I in dependence on the angular frequency of PVDF/1 wt % b-MWCNTs + 3 wt % CB composites, measured at different temperatures.

For PVDF composites filled with 1 wt % b-MWCNTs and 3 wt % CB, the influence of the take-off velocity on electrical properties and thickness was studied. For this purpose, composites were prepared with the two different types of PVDF. As shown in Figure 1, as well as in Figures S1 and S3, the melt viscosity and elasticity of PVDF1 and PVDF2 vary. In order to achieve similar melt viscosities for film extrusion, rheological investigations at different temperatures were carried out on the compounded composite materials, which are here shown for the composite with 1 wt % b-MWCNTs + 3 wt % CB. Due to the higher viscosity of PVDF2, its composite was also investigated at higher temperatures. It could be shown that similar melt viscosities are achieved when the PVDF2-based composite is processed at 280–290 °C, while the PVDF1-based composite is processed between 250 and 270 °C (Figure 6). However, the elasticity level is much higher for the composites as compared to PVDF. As seen in Figures S1 and S3, the pure PVDFs show higher loss moduli G″ than storage moduli G′, with higher values of G′ for PVDF2 than PVDF1. In the composites, as shown in Figures S2 and S3, the elasticity is dominant, as the curves show higher G′ values than G″ and only a low dependence on frequency in the measured range. As a result of these rheological investigations, the PVDF1 composite was extruded into a film at 260–270 °C and the PVDF2 composite was extruded into a film at 280–290 °C.

**Figure 7:**
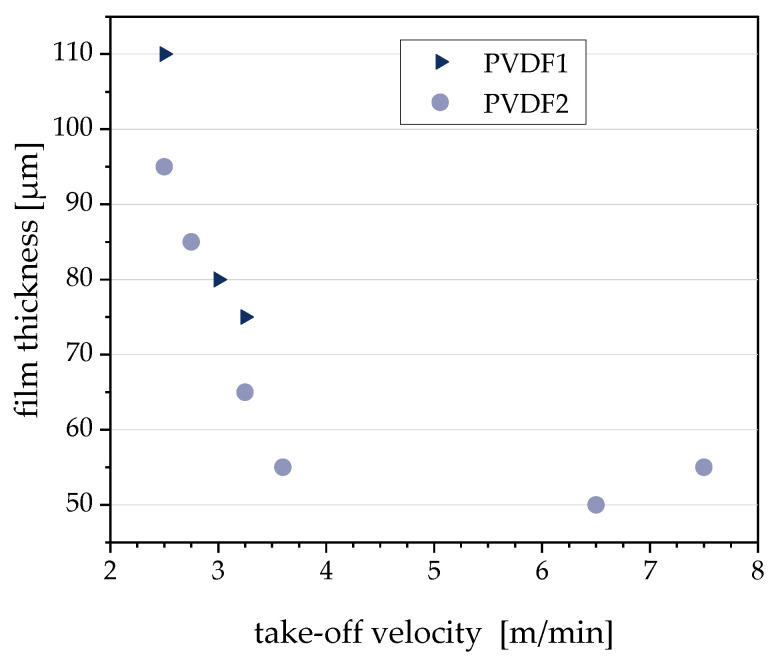
Film thickness in dependence on take-off velocity during film extrusion for PVDF/1 wt % b-MWCNTs + 3 wt % CB composites.

Figure 7 shows the dependence of the film thickness on the take-off velocity during film extrusion. Between 2.5 and 3.6 m/min, the thickness decreases almost linearly with the take-off velocity. However, a further increase of the take-off velocity does not lead to thinner films. The thickness levels off at 50 µm, which seems to be a lower limit for the conditions used. There are differences between the film thicknesses based on PVDF1 and PVDF2, e.g., at 2.5 m/min and 3.6 m/min, the film based on PVDF2 could be drawn to a lower thicknesses compared to that based on PVDF1. With a further increase of the take-off velocity, small holes appeared, which led to tearing of the film. This limit was reached for the PVDF1-based composite above 3.6 m/min and for that based on PVDF2 above 7.5 m/min, showing the better suitability of the PVDF2-based composite material.

**Figure 8:**
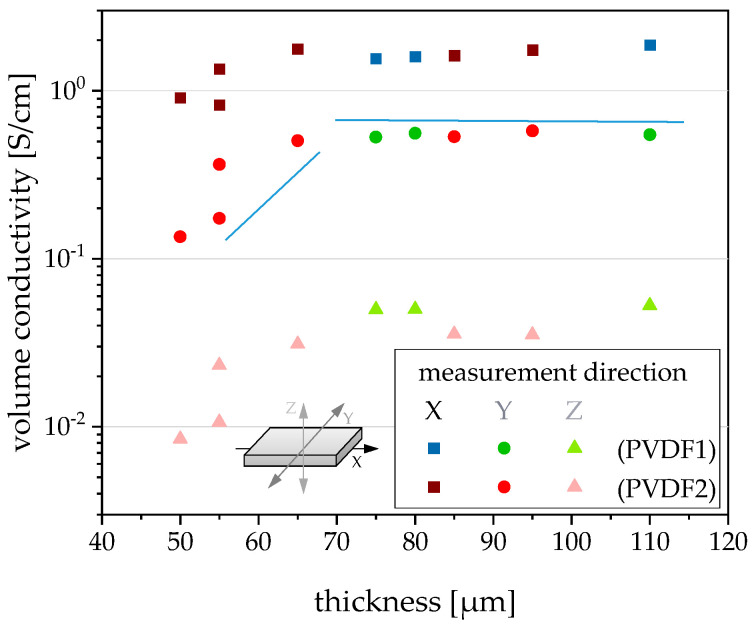
Direction-dependent volume conductivity in dependence on film thickness for PVDF/1 wt % b-MWCNTs + 3 wt % CB composites (blue lines are only for guiding the eyes).

**Figure 9:**
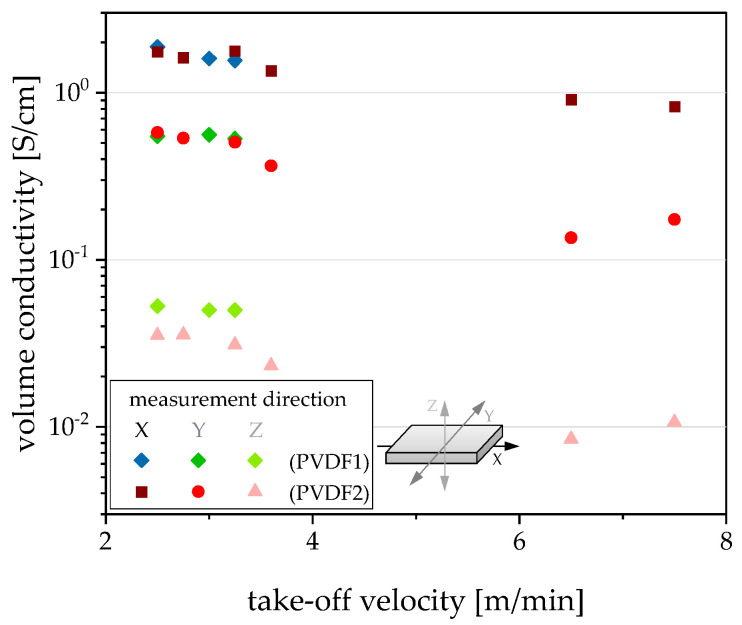
Direction-dependent electrical volume conductivity in dependence on take-off velocity during film extrusion for PVDF/1 wt % b-MWCNTs + 3 wt % CB composites.

Regarding the electrical volume conductivity of films, the values of the x-, y-, and z-direction are nearly in the same range for films with thicknesses varying between 65 and 110 µm (Figure 8). Especially for the x- and y-values of conductivity, the type of PVDF is without significance. For the z-conductivity, the influence of melt viscosity is detectable. The z-conductivities of films are higher if the lower viscous PVDF1 was used as matrix. This correlation also becomes clear when the conductivities are plotted against the take-off velocity (Figure 9). Such a correlation between the matrix viscosity and the electrical conductivity of the composite materials has already been described in the literature [17,18,42,43], however, not for films and mostly for measurements in the in-pane direction.

For films in the thickness range of 50–65 µm, the conductivity values in all three directions are strongly dependent on the film thickness. The thinner the film, the lower the conductivity values. An explanation can be found by plotting the conductivity versus the take-off velocity (Figure 9). The lowest conductivities were measured when the film was extruded using high elongation stresses due to the high take-off velocity. This means that the filler network is stretched considerably, thus reducing the number of filler contact points, leading to a reduced conductivity. The stretched state of the network is frozen by the rapid cooling of the film when it has left the die.

This principle of changing the conductive network structure by stretching upon strain loading of conductive polymer composites is used in resistive strain sensors, where the relationship between conductivity/resistivity and strain is used to measure the deformation of the composite [31,44,45,46].

## 4. Conclusions

For PVDF composites with single fillers such as CNTs or CB and composites with hybrid filler systems of CNTs and CB, direction-dependent electrical properties were characterized on compression-molded plates and extruded films. The mechanical properties of the films were additionally measured. Starting with the recipe development in small-scale melt compounding, the composite preparation was investigated on a laboratory-scale level. It was found that the production route via the direct incorporation of fillers resulted in slightly higher conductivity values of compression-molded plates compared to the masterbatch dilution.

The electrical measurements on compression-molded plates indicated a strong CNT orientation in plane as deduced from the 56–84 times higher conductivity values in plane (x = y) than through plane (z). In contrast, the conductivity ratio σ_x=y/z_ for PVDF/CB composites was only 5-6. In extruded films of PVDF/CNT composites, the conductivity values were 4 times lower perpendicular to the extrusion direction (y), which points to a significant filler orientation in the extrusion direction (x). The in-plane filler orientation (x, y) was more pronounced in the films compared to the compression-molded plates. The aim of the use of hybrid filler systems consisting of CNT and CB was to reach higher conductivity through the film (z) and lower conductivity ratios in plane compared to through-plane. The conductivity ratios relating values in different directions showed that this goal has been achieved, meaning that the overall filler orientation in summary is lower and ranges between the PVDF/CNT and PVDF/CB composites.

The tensile tests of extruded PVDF composite films indicate higher tensile strength, modulus, and elongation at break in extrusion direction as compared to perpendicular to it.

Different films thicknesses were produced by variation of the take-off velocity. At low take-off velocity, the thickness decreases almost linearly with the velocity and a further increase of take-off velocity up to 7.5 m/min does not result in films thinner than 50 µm. The volume conductivities of the films in all directions were nearly independent of the take-off velocity for films with thicknesses between 65 and 110 µm. With thinner films, a slight decrease in volume conductivities could be observed with decreasing film thickness. This is induced by higher take-off velocities, which results in improved stretching of the conductive network structure combined with the loss of electrical contact points. With the selected formulation of PVDF1/1 wt % b-MWCNTs + 3 wt % CB, extruded films with 65 µm thickness and electrical volume conductivities of 1.3 S/cm in the x-direction, 0.39 S/cm in the y-direction, and 0.049 S/cm in the z-direction could be achieved in high quality.

## Figures and Tables

**Table 1:** Comparison of the electrical conductivities of laboratory-scale PVDF1/carbon filler composites shaped to plates (thickness 0.3 mm) and extruded films (thickness 0.1 mm, film extrusion temperature of 225 °C, take-off velocity at 2.5 m/min) and conductivity quotients σ plate/film in x = y (for plates) or x, y (for films), and z directions.

Filler Content	σ_z_ [S/cm]		σ_x = y_ [S/cm]	σ_x_ [S/cm]	σ_y_ [S/cm]	σ_z plate/film_	σ_x = y plate/x film_	σ_x = y plate/y film_
	Plates	Films	Plates	Films	Films			
1 wt % b-MWCNT [26]	1.8 × 10^−2^	3.2 × 10^−3^	1.0 × 10^0^	5.4 × 10^−1^	1.4 × 10^−1^	6	2	7
2 wt % b-MWCNT [26]	2.7 × 10^−2^	1.7 × 10^−2^	2.3 × 10^0^	2.5 × 10^0^	6.9 × 10^−1^	2	1	3
4 wt % CB [26]	2.9 × 10^−2^	6.1 × 10^−2^	1.7 × 10^−1^	2.8 × 10^−1^	2.4 × 10^−1^	0.5	1	1
5 wt % CB	5.0 × 10^−2^	1.1 × 10^−1^	2.4 × 10^−1^	2.7 × 10^−1^	2.8 × 10^−1^	0.5	1	1
1 wt % b-MWCNT+ 1 wt % CB	1.5 × 10^−2^	8.0 × 10^−3^	7.1 × 10^−1^	2.9 × 10^−1^	1.2 × 10^−1^	2	2	6
1 wt % b-MWCNT+ 2 wt % CB	3.7 × 10^−2^	2.8 × 10^−2^	1.6 × 10^0^	1.1 × 10^0^	3.1 × 10^−1^	1	1	5
1 wt % b-MWCNT+ 3 wt % CB	2.4 × 10^−2^	4.9 × 10^−2^	1.4 × 10^0^	1.3 × 10^0^	3.9 × 10^−1^	0.5	1	4
1 wt % b-MWCNT+ 4 wt % CB	2.4 × 10^−2^	6.9 × 10^−2^	1.4 × 10^0^	1.2 × 10^0^	5.6 × 10^−1^	0.3	1	3

**Table 2:** Comparison of conductivity (σ) quotients measured in two and three directions for compression-molded plates and extruded films (film extrusion temperature of 225 °C, take-off velocity at 2.5 m/min).

Filler Content	Plates	Extruded Films		
	σ_x=y/z_	σ_x/z_	σ_y/z_	σ_x/y_
1 wt % b-MWCNT [26]	56	166	42	4
2 wt % b-MWCNT [26]	84	144	40	4
4 wt % CB [26]	6	5	4	1
5 wt % CB	5	3	3	1
1 wt % b-MWCNT+ 1 wt % CB	47	37	15	2
1 wt % b-MWCNT+ 2 wt % CB	43	39	11	3
1 wt % b-MWCNT+ 3 wt % CB	58	26	8	3
1 wt % b-MWCNT+ 4 wt % CB	58	17	8	2

**Table 3:** Tensile test results of extruded films based on PVDF1 composites.

Filler	Thickness [µm]	E_t_ [MPa]	σ_B_ [MPa]	ε_B_ [%]
unfilled	100	2271 ± 118 (x)	48.3 ± 0.4 (x)	60–>100 (x)
		2333 ± 48 (y)	48.9 ± 1.1 (y)	5.4 ± 1 (y)
2 wt % b-MWCNT	100	2673 ± 130 (x)	47.2 ± 2.3 (x)	2.7 ± 0 (x)
		2021 ± 45 (y)	28.0 ± 0.6 (y)	2.3 ± 0 (y)
1 wt % b-MWCNT + 1 wt % CB	100	2441 ± 143 (x)	52.3 ± 1.4 (x)	7.0 ± 1 (x)
		2278 ± 90 (y)	45.1 ± 1.3 (y)	5.1 ± 1 (y)
1 wt % b-MWCNT + 3 wt % CB	100	2230 ± 13 (x)	46.7 ± 0.6 (x)	5.8 ± 0 (x)
		2356 ± 116 (y)	43.0 ± 1.2 (y)	4.2 ± 0 (y)
1 wt % b-MWCNT + 3 wt % CB	65	2105 ± 158 (x)	39.4 ± 2.5 (x)	3.9 ± 1 (x)
		1557 ± 518 (y)	30.8 ± 3.0 (y)	4.1 ± 1 (y)

^1^ E_t_ = E modulus, σ_B_ = tensile strength, ε_B_ = elongation at break; x-direction (parallel to extrusion direction), y-direction (perpendicular to extrusion direction).

## Supplementary Materials

The following are available online at https://www.mdpi.com/2073-4360/12/12/2992/s1, Figure S1: Storage modulus G′ and loss modulus G″ in dependence on the angular frequency of pure PVDF1 and PVDF2, measured at different temperatures, Figure S2: Storage modulus G′ and loss modulus G″ in dependence on the angular frequency of PVDF/1 wt % b-MWCNTs + 3 wt % CB composites, measured at different temperatures, Figure S3: Storage modulus G′ versus loss modulus G″ from frequency sweeps as shown in Figures S1 and S2 for pure PVDF1 and PVDF 2 and their composites with 1 wt % b-MWCNTs + 3 wt % CB, measured at different temperatures, Table S1: DSC data of PVDF1 composites prepared as extruded films.
