# Supplementary material for: Effect of Filler Synergy and Cast Film Extrusion Parameters on Extrudability and Direction-Dependent Conductivity of PVDF/Carbon Nanotube/Carbon Black Composites
Source: Polymers (Basel). 2020 Dec 15;12(12):2992. doi: 10.3390/polym12122992 (PMC7765291; doi:10.3390/polym12122992)
Supplement: Supplementary file 1 [file polymers-12-02992-s001.pdf]

Article

Effect of filler synergy and cast film extrusion parameters on extrudability and direction-dependent conductivity of PVDF/carbon nanotube/carbon black composites

Beate Krause [1], Karina Kunz[1], Bernd Kretzschmar [1], Ines Kühnert [1] and Petra Pötschke [1,*]

Supplementary materials

Supplementary Materials: The following are available online at www.mdpi.com/xxx/s1, Figure S1: Storage modulus G′ and loss modulus G′′ in dependence on the angular frequency of pure PVDF1 and PVDF2, measured at different temperatures, Figure S2: Storage modulus G′ and loss modulus G′′ in dependence on the angular frequency of PVDF/1 wt% b-MWCNTs + 3 wt% CB composites, measured at different temperatures, Figure S3: Storage modulus G′ versus loss modulus G′′ from frequency sweeps as shown in Figs. S1 and S2 for pure PVDF1 and PVDF 2 and their composites with 1 wt% b-MWCNTs + 3 wt% CB, measured at different temperatures, Table S1: DSC data of PVDF1 composites prepared as extruded film.

1. Additional results of melt rheological characterization

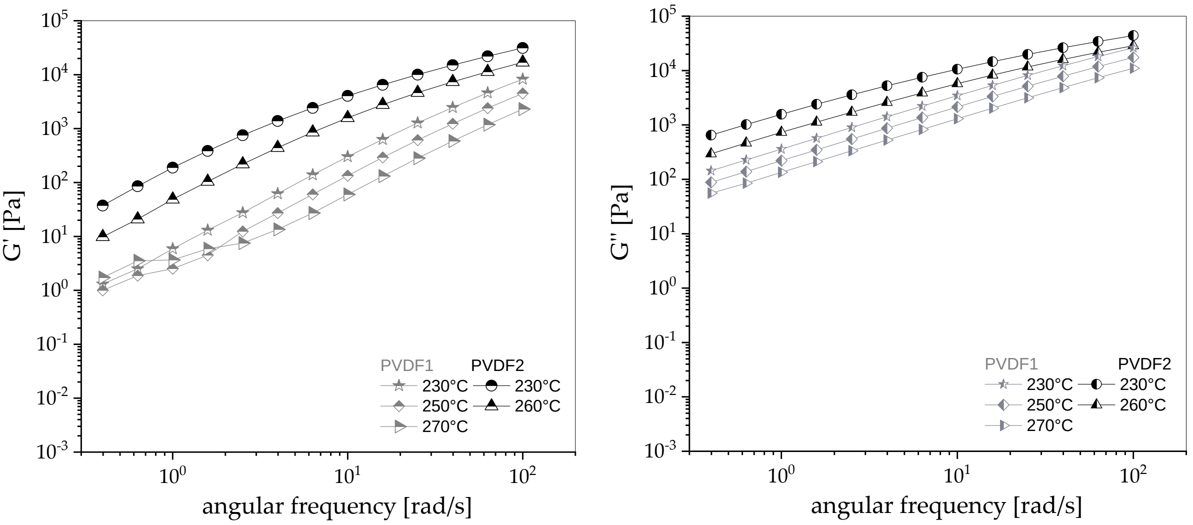

Figure S1: Storage modulus G′ and loss modulus G′′ in dependence on the angular frequency of pure PVDF1 and PVDF2, measured at different temperatures.

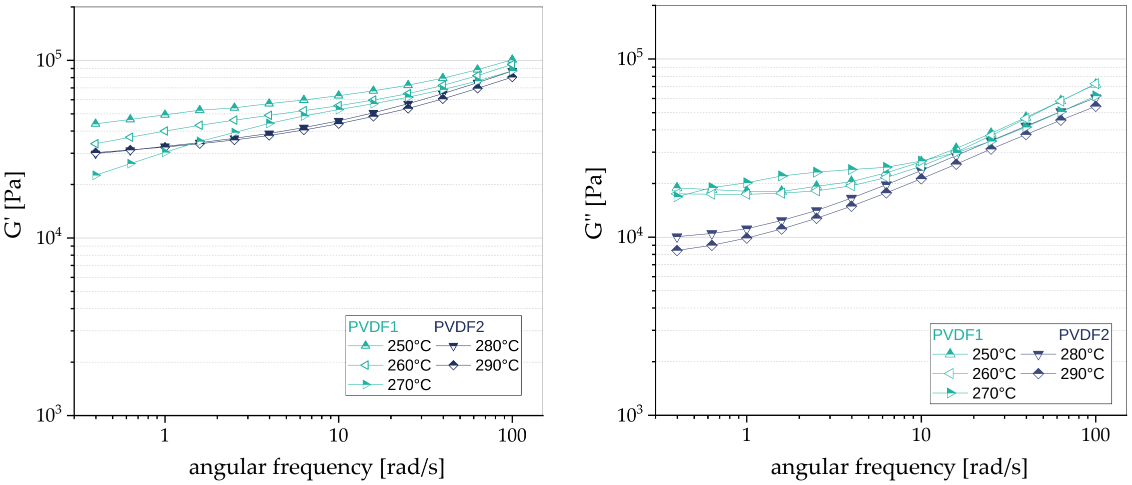

Figure S2: Storage modulus G′ and loss modulus G′′ in dependence on the angular frequency of PVDF/1 wt% b-MWCNTs + 3 wt% CB composites, measured at different temperatures.

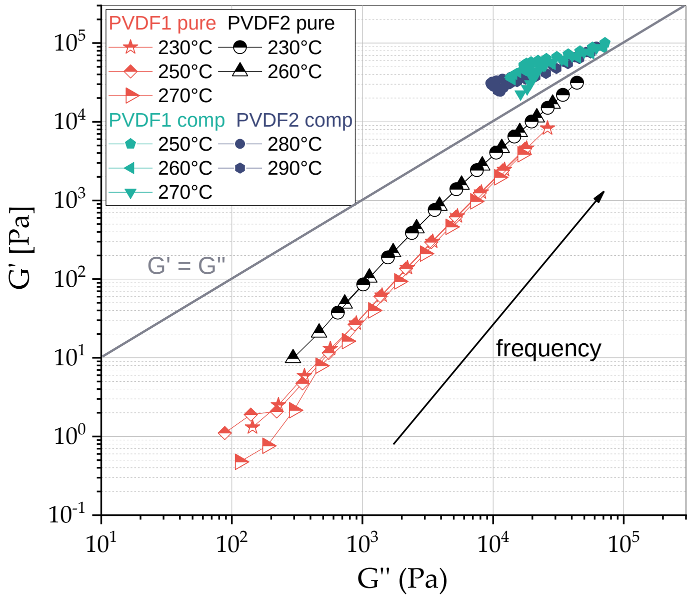

Figure S3: Storage modulus G′ versus loss modulus G′′ from frequency sweeps as shown in Figs. S1 and S2 for pure PVDF1 and PVDF 2 and their composites with 1 wt% b-MWCNTs + 3 wt% CB, measured at different temperatures.

2. Dynamic scanning calorimetry (DSC) results of different film samples

Table S1. DSC results of PVDF1 composites prepared as extruded films.

filler	Thickness [μm]	ΔH_m[1] [J/g PVDF]	T_m [°C] [2]	$T_{c, max}$ [°C]	$T_{c, onset}$ [°C]
Unfilled PVDF1	100	75.8	169.3; 172.9	63.4; 140.3	143.1
1 wt% b-MWCNT	100	72.7	171.8; 176.0s	65.7; 150.8	152.4
2 wt% b-MWCNT	100	73.6	172.1; 176.5s	65.7; 151.6	152.8
1 wt% b-MWCNT + 1 wt% CB	100	73.5	171.8; 177.0s	65.7; 150.8	152.1
1 wt% b-MWCNT + 2 wt% CB	100	75.5	172.0	65.7; 151.1	152.2
1 wt% b-MWCNT + 3 wt% CB	100	71.3	172.1	65.1; 150.9	152.2
1 wt% b-MWCNT + 3 wt% CB	80	71.4	172.2	65.0; 150.9	152.3
1 wt% b-MWCNT + 3 wt% CB	70	71.6	172.0	65.0; 150.9	152.2
4 wt% CB	100	70.0	172.0	65.7; 149.2	150.6

[1] ΔH calculated from 2. heating run

[2] "s" indicates a shoulder in the melting curve

Differential scanning calorimetry (DSC) was performed to characterize the thermal behavior of unfilled polymer and composites using a Q 2000 (TA instruments, New Castle, DE, USA) under nitrogen atmosphere in a temperature range of -80°C to 200°C, with a cooling/heating rate of 10 K/min and a run cycle of 1st heating–cooling–2nd heating.. $\mathbf{\Delta H_m}$ is the melting enthalpy obtained in the second heating run, $\mathbf{T_m}$ is the melting temperature in the second heating run, $\mathbf{T_{c, max}}$ the temperature of the maximum in the crystallization curve and $\mathbf{T_{c, onset}}$ the onset temperature of crystallization.
